# Polyether Demulsifier Complexes for Efficient Demulsification of Water-in-Heavy Oil Emulsions

**DOI:** 10.3390/molecules30122550

**Published:** 2025-06-11

**Authors:** Jing Li, Xiao Xia, Jinlong Gao, Hao Chen, Jun Ma

**Affiliations:** 1 Department of Chemical Engineering, Textile and Clothing, Shaanxi Polytechnic Institute, Xianyang 712000, China; 20131283@sxpi.edu.cn (J.L.); 20201534@sxpi.edu.cn (J.G.); 2 Department of Chemical Engineering, School of Chemistry and Chemical Engineering, Guizhou University, Guiyang 550025, China; xiaxiao2021@163.com; 3 China Tianchen Engineering Corporation, Tianjin 300400, China; chenhao@cntcc.cn

**Keywords:** polyether demulsifier complexes, interfacially active asphaltenes, demulsification, water-in-heavy oil emulsions

## Abstract

In the production process of the heavy oil industry, efficiently demulsifying water-in-heavy oil (W/HO) emulsions can effectively prevent the negative effects of emulsion corrosion on equipment, increase costs, reduce oil quality, and pollute the environment. Herein, polyether demulsifier complexes (PDC) were obtained by compounding fatty alcohol nonionic polyether (FAP) with perfluoropolyether (PFPEA, [CF_3_O(CF_2_CF_2_O)_n_CF_3_]) through a simple physical blending method. The experimental results demonstrate that PDC exhibited outstanding demulsification performance for W/HO emulsions across varying temperatures: At 60 °C and 400 ppm dosage, PDC achieved complete dehydration (100%) within just 2 min, showing significantly faster demulsification kinetics compared to FAP and PFPEA. Even at the reduced temperature of 40 °C, PDC maintained effective demulsification capability, achieving complete phase separation within 6 min. These findings collectively establish PDC’s superior demulsification efficiency for W/HO emulsions, with particularly remarkable performance under challenging low-temperature conditions. Research on the demulsification mechanism indicates that PDC achieves efficient demulsification performance due to the synergistic effect the synergistic effect of FAP and PFPEA to effectively destroy the non-covalent bonds (hydrogen and π–π stacking) of interfacially active asphaltenes (IAA) at the oil–water interface, thereby achieving demulsification of W/HO emulsion. PDC with outstanding demulsification ability exhibits significant potential for practical applications in heavy crude oil–water emulsion treatment, and this work can provide insights for developing new composite demulsifiers for petroleum production.

## 1. Introduction

Heavy oil, an important component of petroleum resources, occupies a significant position in the energy sector [1]. However, in the heavy oil industry, the formation of water-in-heavy-oil (W/HO) emulsions is inevitable during the separation of heavy oil from oil ores due to two main factors: the presence of natural interfacial active components (e.g., asphaltenes, resins, naphthenic acids, and amphiphilic solid particles) in heavy oil [2], and the involvement of water [3]. These emulsions can lead to serious problems, including equipment corrosion, reduced oil quality, and environmental pollution [4,5,6]. Therefore, achieving efficient demulsification of W/HO emulsions is necessary for the downstream processing of heavy oil.

Understanding the stabilization mechanism of W/HO emulsions is a prerequisite for effective demulsification. Research has shown that asphaltenes are the main stabilizers of W/HO emulsions, consisting of aromatic rings, aliphatic chains, heteroatoms (S, N, O), and trace heavy metal elements (Fe, V, Ni) [7]. However, Yang et al. [8] found that only about 2 wt% of interfacially active asphaltenes (IAA) within the asphaltene play a dominant role in stabilizing W/HO emulsions. Previous studies have demonstrated that IAA could be adsorbed at the oil–water interface by non-covalent forces (hydrogen bonds, π–π stacking), forming a certain thickness of interfacial films and preventing water droplets from aggregation and coalescence [9]. Therefore, the key to realizing demulsification of W/HO emulsions lies in disrupting the IAA interfacial films.

Currently, chemical demulsification is widely employed in the petroleum industry due to its high efficiency, cost-effectiveness, and broad applicability [10,11,12]. The key to this method depends on chemical demulsifiers, which effectively destabilize the IAA interfacial film. For instance, we previously synthesized a nonionic polyether (JMNP) with oxygen-containing functional groups such as hydroxyl and ester groups [13]. Experiments demonstrated a dehydration rate of up to 100% under conditions of 60 °C for 30 min at a concentration of 400 ppm. Subsequently, we developed another oxygen-enriched nonionic polyether (MJTJU-2), which contains oxygen-containing groups like hydroxyl, ester, carboxyl, and ether groups [14]. At a dosage of 400 mg/L, a temperature of 60 °C, and a demulsification time of 15 min, the demulsification efficiency reached 97%. Research indicates that the oxygen-containing groups in nonionic polyethers can effectively disrupt the non-covalent interactions (hydrogen bonds and π–π stacking) of IAA at the oil–water interface, form new hydrogen bonds with water molecules, and thereby enhance oil–water separation. Zhang et al. [15] synthesized a network-branched fluorinated polyether (RBF) using block polymerization. At a dosage of 50 mg/L, 60 °C, and a demulsification time of 2 h, the demulsification efficiency for W/O emulsions reached 95.64%. This high efficiency is attributed to the network-branched structure of RBF, which enhances interfacial film displacement and promotes water droplet coalescence. However, most previous studies have focused on single-component demulsifiers, which often require high temperatures and long demulsification times. Research on composite demulsifiers for W/HO emulsions has rarely been reported. Given that the electronegativity of F atoms is stronger than that of O atoms and can form stronger hydrogen bonds with water molecules, combined with the principle of synergistic effect, the combination of oxygen-containing polyether and fluorine-containing polyether is expected to develop highly efficient composite demulsifiers, achieving efficient demulsification of W/HO emulsions.

To sum up, to achieve efficient demulsification of W/HO emulsions, this study first combined fatty alcohol nonionic polyether (FAP) and perfluoropolyether (PFPEA, [CF_3_O(CF_2_CF_2_O)_n_CF_3_]) through a simple physical blending method, resulting in polyether demulsifier complexes (PDC). The demulsification performance of the PDC in the IAA-stabilized W/HO emulsions was then evaluated using the standard bottle test method. Finally, the demulsification mechanism of the PDC was investigated through interfacial characterization and molecular dynamics simulations. These findings provide a theoretical foundation and practical guidance for the large-scale industrial application of composite polyether demulsifiers.

## 2. Experimental Section

### 2.1. Chemicals and Samples

All chemical reagents were analytical grade and directly used without further purification. Toluene was purchased from Guizhou Hangmao Chemical Co., Ltd.(Guiyang, China). Fatty alcohol nonionic polyether (FAP) and perfluoropolyether (PFPEA) were purchased from Nantong Derek Chemical Co., Ltd. (Nantong, China). The milli-Q water (with a resistance of 18.2 MΩ·cm at 25 °C, Millipore, Burlington, MA, USA) was used throughout this study. The heavy oil samples were extracted from Indonesian asphalt rocks by toluene and heptane. The experimental procedure could be found elsewhere [16,17]. The interfacial active asphaltenes (IAA) were extracted using the procedures given elsewhere [3].

### 2.2. Characterizations of Demulsifiers

Molecular weight analysis was performed on an Agilent GPC 50 system (Santa Clara, CA, USA) equipped with a refractive index detector. The system was calibrated with polystyrene standards (M_w_ range: 500–500,000 g/mol) using tetrahydrofuran (HPLC grade) as the eluent at 30 °C. The functional group composition of the demulsifier was comprehensively characterized using Fourier transform infrared spectroscopy (FTIR; FTS6000, Bio-Rad, Hercules, CA, USA) and nuclear magnetic resonance spectroscopy (NMR; AVANCE III, Bruker, Billerica, MA, USA). FTIR analysis was performed at ambient temperature (25 ± 1 °C) with spectra recorded in the 4000–400 cm⁻^1^ range. ^1^H NMR spectra were acquired at 25 °C in deuterated chloroform (CDCl_3_) using tetramethylsilane (TMS) as internal reference, with a 90° pulse width.

The surface tension was conducted at 25.0 ± 0.5 °C, and the interfacial tension was performed at 60.0 ± 0.5 °C using a tensiometer (FCA2000A4R) with a 1 mL high-precision glass syringe, where the integrated software (FCA2000A4R) analyzed droplet images.

### 2.3. Demulsification Performance Test

The demulsification performance of PDC in W/O emulsions was evaluated by the bottle test method according to the standard procedures specified in ASTM D2711-17 and SY/T 5281-2000. The emulsions were prepared according to the following procedures to evaluate the ability of different demulsifiers in W/HO emulsions. A total of 100 mL IAA-in-toluene solution (10 g/L) was employed with 10 mL of ultrapure water using a homogenizer at 15,000 rpm for 5 min. The obtained W/HO emulsions were kept steady for over 12 h to reach the equilibrium state. Then, the 7.5 mL emulsion phase was removed and put in a stoppered graduated cylinder with a minimum scale of 0.5 mL. The different demulsifiers were added to the emulsions at a certain concentration. The cylinder was shaken for 2 min to make the demulsifier and the emulsions well-mixed. The mixture was placed in a constant temperature water bath for oil–water separation. The emulsion without demulsifier was employed as a reference (blank). The demulsification efficiency was evaluated by dehydration ratio. The dehydration ratio was calculated based on Formula (1) [18].

(1) $$\;\mathrm{Dehydration}\;\mathrm{ratio}=\frac{V_{t}}{V_{0}}\;\times \;100\%\;$$

where Vt is the separated water volume measured by a stoppered graduated cylinder, and V_0_ represents the initial water volume (10 mL). Volume measurements were conducted using the meniscus bottom alignment method. In cases where the oil–water interface exhibited irregular morphology, triplicate readings were taken at 120° intervals and averaged to ensure measurement accuracy. In addition, we also used a syringe to measure the water extracted each time, in order to verify and obtain accurate values. For each experimental group, three independent replicates were conducted, and the average value was calculated.

### 2.4. Molecular Dynamics Simulation

**Figure 1:**
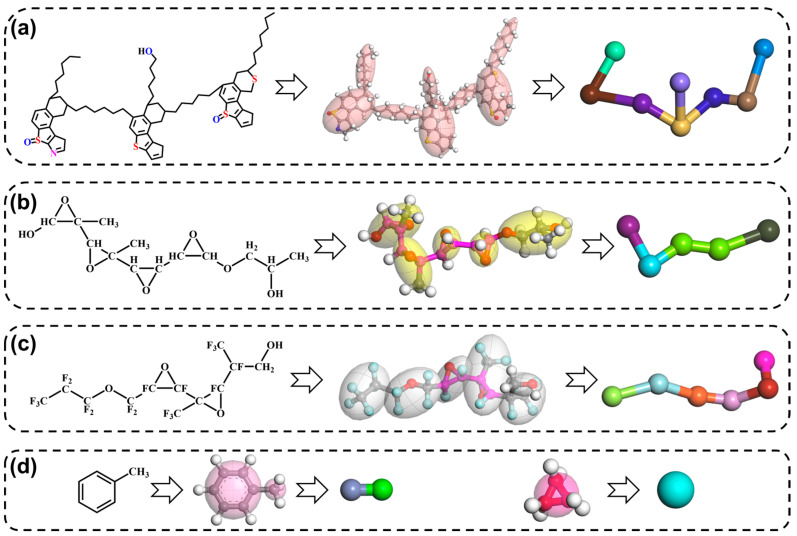
The selected and constructed calculation models and coarse-graining models of different molecular structures ((**a**): IAA, (**b**): FAP demulsifier, (**c**): PFPEA demulsifier, (**d**): toluene and water).

Selection and construction of calculation model: The selected and constructed calculation models are water, toluene, IAA, FAP, PFPEA, the oil–water interface model (Figure S1a), and the mesoscopic molecular model (Figure S1b). These structures of geometry optimization by Materials Studio 8.0 software are shown in Figure 1. The dissipative particle dynamics (DPD) simulation technique in the Materials Studio software has been used to study the demulsification process of the W/HO emulsions. The primary step of using DPD to perform simulation studies is the coarse-graining of the molecular structure [19,20,21]. In this step, the selected molecular structure models were coarse-grained with different beads. Moreover, according to our previous research results [22], the IAA molecule was replaced with 8 beads, and the toluene molecule was replaced with 2 beads. The three water molecules were replaced with one bead [20]. In addition, to shorten the calculation time and improve the calculation efficiency, the FAP demulsifier molecule was replaced with 5 beads. The PFPEA demulsifier molecule was replaced with 6 beads. The atomic clusters represented by each bead, and repulsive force parameters between the beads are shown in Tables S1–S3. The repulsive force parameters between the beads were calculated by the blends module in the Materials Studio software [19]. The energy change curves of the above structural models in the process of geometric structure optimization are shown in Figure S2.

All-atom simulation calculation details: All-atom molecular dynamics simulation calculation was implemented by the Forcite module of Materials Studio. Calculation accuracy was chosen as ultra-fine. Molecular dynamic (MD) simulation was carried out at NVT-ensemble (constant number of atoms (N), volume (V), and temperature (T) for the established sorption system). Temperature and pressure were set at 298 K and 1 atm, respectively, using the algorithm described by Ma et al. (2021) [22]. The COMPASS II was selected as the force field. Van der Waals and electrostatic interaction were all based on group-based; cut off distance was 1.85 nm. The dynamic time and time step were 500 ps and 1 fs, respectively. The system was at equilibrium when the energy and temperature fluctuated around a constant value.

Dissipative particle dynamic (DPD) simulation details: The DPD simulations were carried out in the Mesocite module of Materials Studio. The simulations were accomplished in a cubic box; the size of the box was 5 × 5 × 5 nm^3^. The number of beads was 1.92 × 10^5^, and the periodic boundary temperature was 295 K in the box. The force field was determined based on the repulsive force parameters between mesoscopic molecules. The DPD simulated time step was set as 10,000 ps.

## 3. Results and Discussion

### 3.1. Characterization and Analysis of Demulsifiers

**Figure 2:**
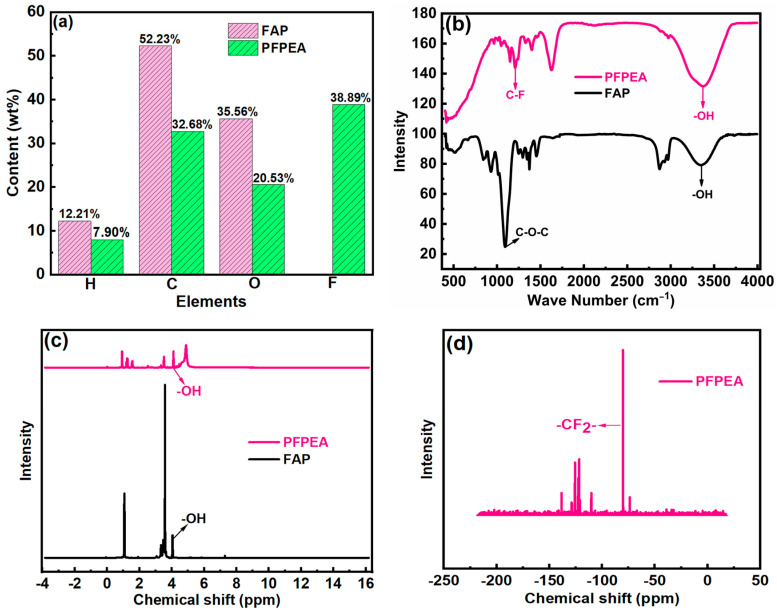
(**a**) Element composition of demulsifier samples; (**b**) FTIR spectra of demulsifier samples; (**c**) ^1^H NMR spectra of demulsifier samples; (**d**) ^19^F NMR spectrum of PFPEA demulsifier.

To characterize the molecular structures of different demulsifiers, we employed GPC, elemental analysis, FTIR, and NMR to determine the molecular weight, elemental composition, and functional groups of FAP and PFPEA. The results are summarized in Table 1 and Figure 2.

As shown in Table 1, FAP exhibited a weight average molecular weight (M_w_) of 5215 g/mol and a number average molecular weight (M_n_) of 3754 g/mol, with a polydispersity index (PDI) of 1.39. In comparison, PFPEA demonstrated significantly lower molecular weights (M_w_ = 1264 g/mol, M_n_ = 985 g/mol) but a narrower molecular weight distribution (PDI = 1.28). Figure 2a depicts the elemental composition of FAP and PFPEA. FAP was composed of H, C, and O elements with contents of 2.21%, 52.23%, and 35.56%, respectively. PFPEA was composed of H, C, O, and F elements with contents of 7.90%, 32.68%, 20.53%, and 38.89%, respectively. Figure 2b presents the FTIR spectra of FAP and PFPEA. The absorption peak at 3400 cm^−1^ was the stretching vibration peak of -OH, the absorption peak at 1092 cm^−1^ was the C-O-C bond in FAP, and the absorption peak at 1204 cm^−1^ was the C-F bond in PFPEA. Figure 2c depicts the ^1^H NMR spectra of FAP and PFPEA, with a signal peak of hydroxyl groups at a chemical shift of 4.06 ppm, indicating the presence of hydroxyl groups in FAP and PFPEA, which corresponded to the results of the FTIR spectra. Figure 2d depicts the ^19^F NMR spectrum of PFPEA, with a typical -CF_2_ signal peak at a chemical shift of −80.118 ppm. These results indicate that the FAP purchased was indeed a nonionic fatty alcohol polyether, and PFPEA was indeed a perfluoropolyether.

### 3.2. Demulsification Performance of PDC in Water-in-Heavy Oil Emulsions

**Figure 3:**
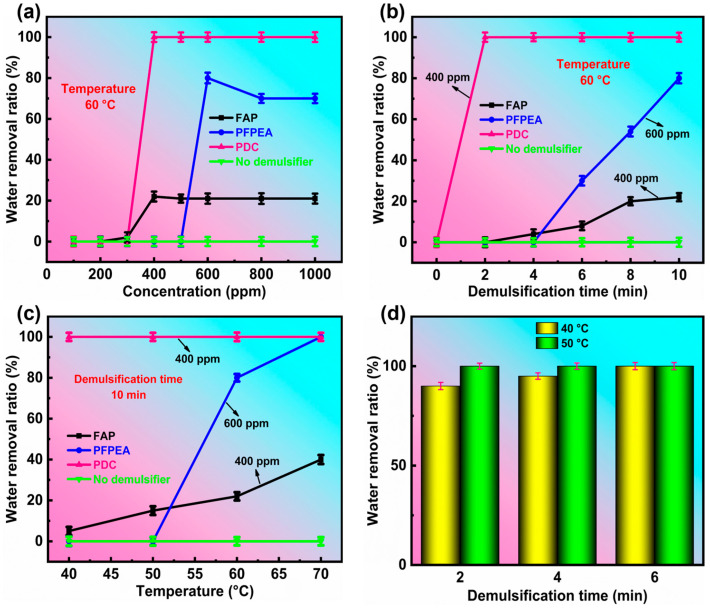
Demulsification performance of demulsifiers in W/HO emulsions. (**a**) The effect of demulsifier concentration on dehydration performance, (**b**) the variation of dehydration ratio with demulsification time, (**c**) the variation of dehydration ratio with demulsification temperature, (**d**) the dehydration rate of PDC at 40 °C and 50 °C.

To test the demulsification effect of the PDC in water-in-heavy oil emulsions, its demulsification performance was evaluated by the standard bottle test method of the petroleum industry. The results are shown in Figure 3. All experiments were conducted in triplicate (*n* = 3) with standard deviations <4.8% for dehydration rates.

**Figure 4:**
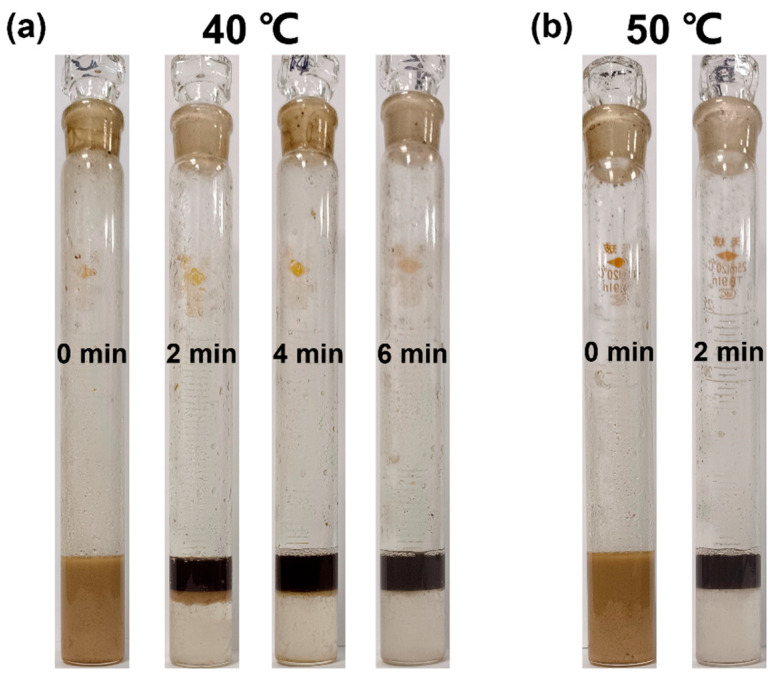
Dehydration photos of PDC demulsified W/HO emulsion at different temperatures: (**a**) 40 °C, (**b**) 50 °C.

Figure 3a depicts the variation of the dehydration ratio with the concentration of different demulsifiers. When the demulsification temperature was 60 °C and the demulsification time was 10 min, the control experiment (without demulsifier) showed no water separation in W/HO emulsions. The optimal demulsification concentrations for FAP, PFPEA, and PDC were 400, 600, and 400 ppm, respectively, achieving water removal efficiencies of 22%, 80%, and 100% after 10 min of treatment. Beyond their respective optimal concentrations, the demulsification performance of FAP and PFPEA was observed to decrease. These results demonstrate the superior performance of the composite demulsifier (PDC) compared to individual components. Figure 3b depicts the change in the dehydration ratio of W/HO emulsions with demulsification time under the optimal demulsification concentration of different demulsifiers. When the demulsification time was 2 min, the PDC completely realized the demulsification of emulsions, while the demulsification efficiency of FAP and PFPEA was 0 at this time. When the demulsification time exceeded 4 min, although the dehydration capacity of PFPEA and FAP was gradually increasing, the demulsification rate was still not as fast as that of the composite demulsifiers. Figure 3c demonstrates the temperature dependence of dehydration efficiency for the investigated demulsifiers. PDC achieved complete phase separation (100% dehydration) within 10 min at 40 °C, while both FAP and PFPEA exhibited temperature-dependent performance enhancements, with dehydration efficiency increasing proportionally with temperature elevation. At elevated temperatures (70 °C), FAP and PFPEA demonstrated significantly enhanced dehydration efficiencies of 40% and 100%, respectively. This temperature-dependent behavior can be attributed to accelerated Brownian motion of demulsifier molecules at the oil–water interface, which facilitates effective disruption of interfacial films [23]. The demulsification performance of PDC was systematically evaluated at lower temperatures (<60 °C) (Figure 3d). Remarkably, PDC exhibited outstanding low-temperature efficiency, achieving 90% water removal within 2 min at 40 °C, which increased to 95% after 4 min and reached complete phase separation (100%) by 6 min. When the temperature was elevated to 50 °C, the system attained complete demulsification (100%) in merely 2 min, demonstrating significantly enhanced performance with moderate heating. These results highlight PDC’s exceptional capability for rapid demulsification even under challenging low-temperature conditions. The dehydration photos are shown in Figure 4, revealing that PDC exhibits outstanding demulsification efficiency for W/HO emulsions at low temperatures, suggesting significant potential for industrial applications. Furthermore, a comparison of the demulsification efficiency of PDC with other single-component demulsifiers [13,14,15,24,25,26,27] (Table 2) demonstrated that PDC is superior. In conclusion, the composite PDC demulsifier, formulated by combining FAP and PFPEA, demonstrated superior demulsification performance for W/HO emulsions compared to either individual component.

### 3.3. Demulsification Mechanism of PDC in W/HO Emulsions

To elucidate the demulsification mechanism of PDC in W/HO emulsions, we combined interfacial characterization techniques with molecular dynamics simulations to investigate PDC’s action mechanism in the emulsions’ demulsification process.

**Figure 5:**
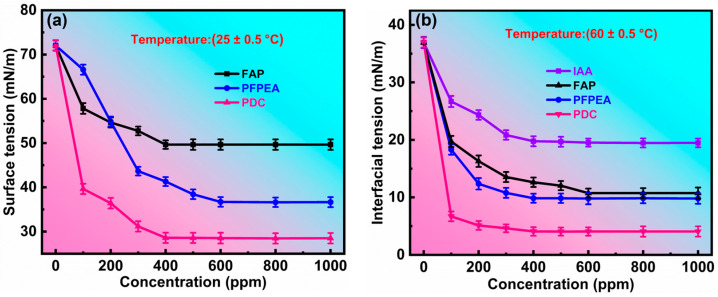
Characterization of interfacial activity of demulsifiers. (**a**) The variation of surface tension with concentration. (**b**) The variation of interfacial tension with concentration.

Our previous research demonstrated that the stability of W/HO emulsions originates from interfacial active asphaltenes (IAA) in heavy oil, forming a viscoelastic interfacial film at the oil–water interface through non-covalent interactions (π–π stacking, hydrogen bonding and van der Waals force), which effectively prevents water droplet aggregation and coalescence, thus stabilizing the emulsions [13]. The surface and interfacial activity of demulsifiers plays a critical role in their ability to rapidly migrate from the oil phase to the oil–water interface and interact with IAA. Consequently, we characterized the surface and interfacial activity of FAP, PFPEA, and PDC. Figure 5 presents the variation curves of surface tension and interfacial tension of air–water and toluene–water phases with the concentration of demulsifiers. All experiments were conducted in triplicate (*n* = 3) with standard deviations <3% for dehydration rates. As shown in Figure 5a, FAP, PFPEA, and PDC effectively reduced both the surface tension of water and the interfacial tension between toluene and water, demonstrating their surface-active and interfacial-active properties. However, significant differences existed in the surface and interfacial activity among FAP, PFPEA, and PDC. At a concentration of 100 ppm in water, FAP, PFPEA, and PDC reduced the surface tension of water from 72.053 mN/m to 57.826 mN/m, 66.582 mN/m, and 39.649 mN/m, respectively. When the concentrations were increased to 400 ppm (FAP), 600 ppm (PFPEA), and 400 ppm (PDC), the surface tensions further decreased to 49.659 mN/m, 36.701 mN/m, and 28.599 mN/m, respectively. The greater reduction in surface tension indicated that PDC possesses superior surface activity compared to FAP and PFPEA. Beyond this concentration, further increases in demulsifier concentration maintained nearly constant water surface tension, indicating attainment of the critical micelle concentration (CMC) and maximum surface activity. As shown in Figure 5b, FAP, PFPEA, and PDC reduced the interfacial tension between toluene and water more effectively than IAA, confirming their stronger interfacial activity than IAA. In addition, at a concentration of 100 ppm in water, FAP, PFPEA, and PDC reduced the interfacial tension of toluene–water from 36.936 mN/m to 19.734 mN/m, 18.325 mN/m, and 6.7 mN/m, respectively. However, at their respective CMC values, FAP, PFPEA, and PDC reduced the toluene–water interfacial tension to 12.632 mN/m, 9.798 mN/m, and 4.066 mN/m, indicating that the interfacial activity of PDC is stronger than FAP and PFPEA. At this point, further increases in demulsifier concentration maintained a nearly constant interfacial tension between toluene and water, indicating that (1) the demulsifier formed micelles at the oil–water interface, (2) its amphiphilic molecular arrangement was altered, and (3) its interfacial activity strength reached a maximum value. In conclusion, the above results indicate that PDC’s superior surface and interfacial activity facilitated its rapid diffusion to the oil–water interface and subsequent interaction with IAA, thereby accelerating the demulsification of W/HO emulsions. Moreover, the results of surface and interfacial tension measurements indicated that the CMC values of FAP, PFPEA, and PDC corresponded to their optimal demulsification concentrations.

**Figure 6:**
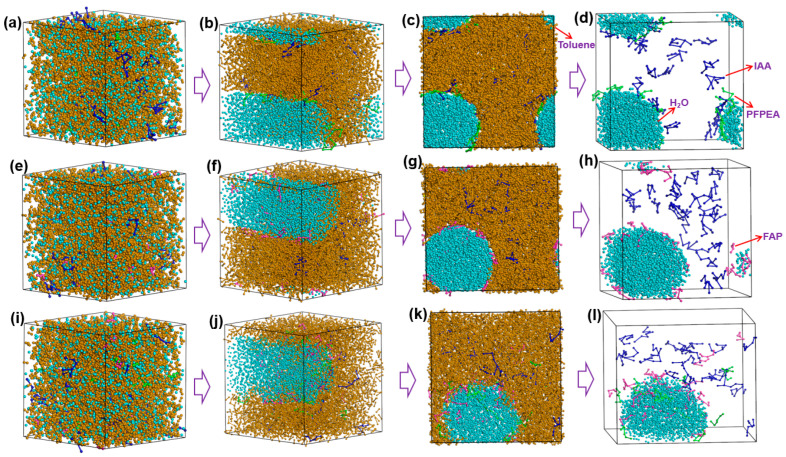
The variation of dynamic demulsification processes of different demulsifiers. (**a**–**d**) PFPEA, (**e**–**h**) FAP, (**i**–**l**) PDC.

**Figure 7:**
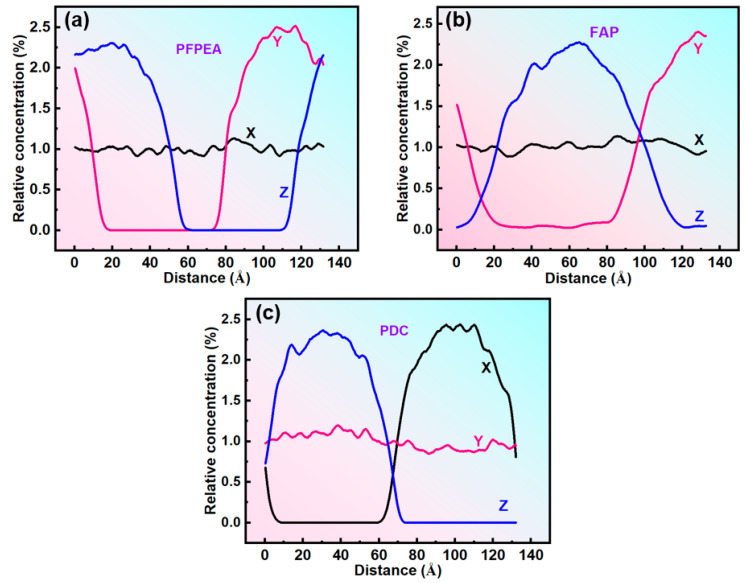
Water concentration variation curve during the DPD simulation process. (**a**) PFPEA, (**b**) FAP, (**c**) PDC.

Dissipative particle dynamics (DPD) simulations were employed to investigate the dynamic demulsification processes of different demulsifiers in W/HO emulsions. All-atom molecular dynamics (MD) simulations were performed to investigate the interfacial replacement processes of IAA at the oil–water interface. Simulation balance verification is also shown in Figures S3 and S4. Furthermore, the DPD simulation results presented in Figure 6 reveal that the demulsification of water-in-heavy oil (W/HO) emulsions occurred through two distinct processes: (1) the water molecule aggregation stage, characterized by rapid coalescence of highly dispersed water molecules into droplets through interactions with demulsifier molecules; (2) the coalescence stage of water droplets, during which water molecules aggregate to form water droplets, ultimately resulting in the separation of oil and water phases under the action of gravity. Furthermore, FAP, PFPEA, and PDC can all promote the aggregation of water molecules, but the dynamic simulation time required for water molecule aggregation is different. The kinetic simulations quantified distinct coalescence times for complete water molecule aggregation: 9000 ps for FAP, 7500 ps for PFPEA, and 4000 ps for PDC. It also reflects that the dehydration ratio of PDC in W/HO emulsions is faster than that of FAP and PFPEA. In addition, Figure 7 depicts the variation of water concentration in the system with distance during the DPD simulation process. It shows that water molecules underwent aggregation during the dynamic demulsification process. Following dynamic demulsification simulations, distinct spatial aggregation patterns emerged: PFPEA induced preferential water molecule accumulation in the OYZ plane, whereas FAP and PDC promoted aggregation primarily in the OXY plane. Furthermore, the demulsifiers adsorbed on the water molecules’ surfaces, and IAA molecules were distributed in the oil phase, indicating that the demulsifier interacted with IAA molecules.

**Figure 8:**
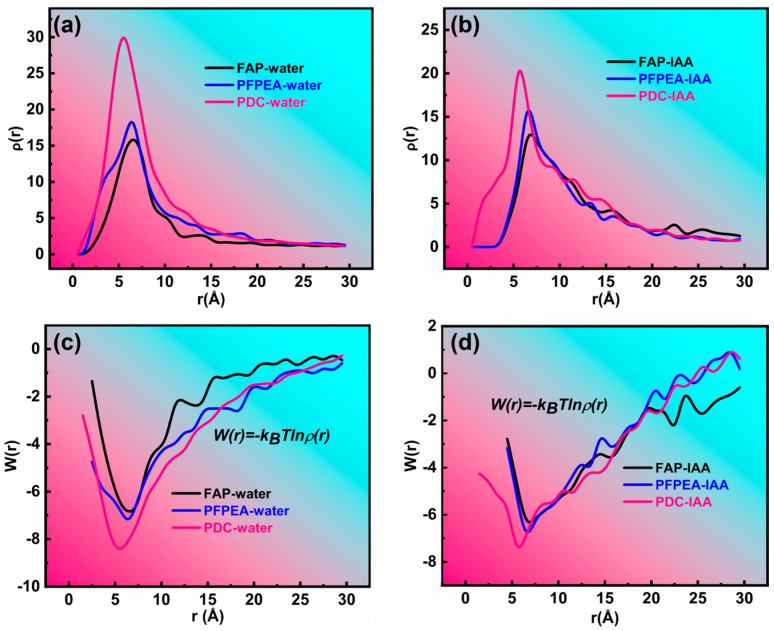
The radial distribution function (**a**,**b**) and potential of mean force (**c**,**d**) of different demulsifiers.

**Figure 9:**
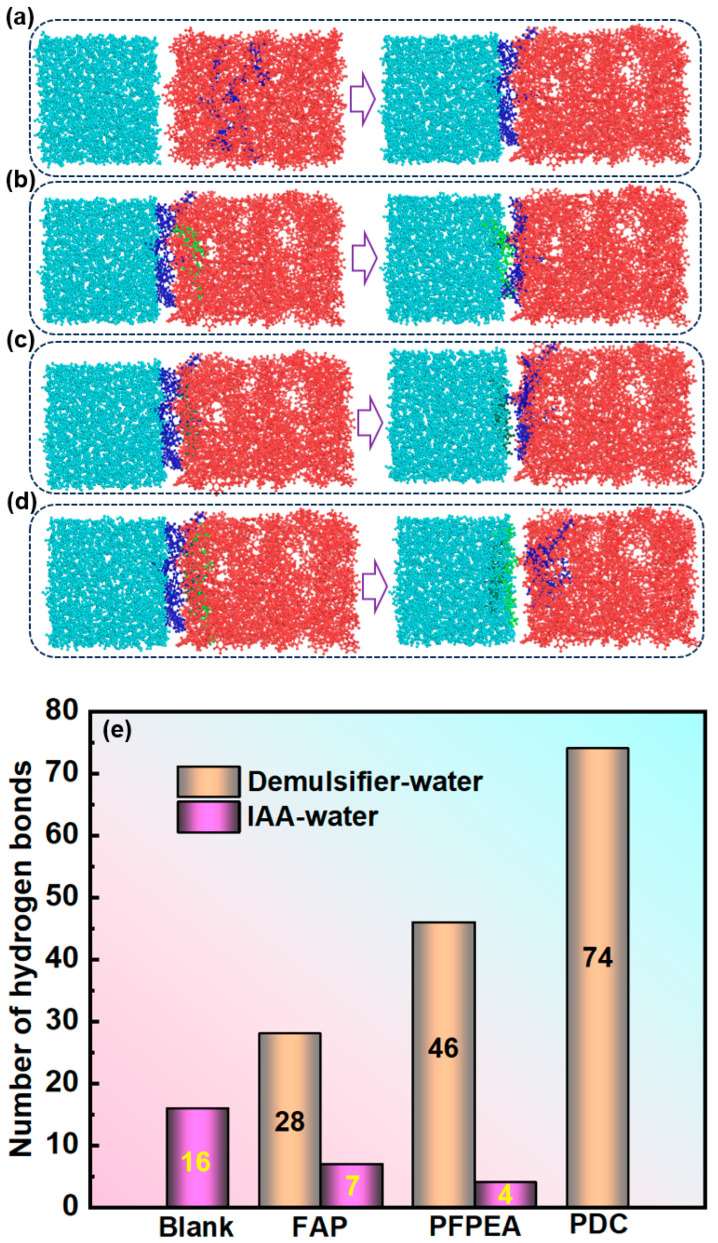
All-atom molecular dynamics simulation results: (**a**) IAA, (**b**) FAP, (**c**) PFPEA, (**d**) PDC. (**e**) Changes in the number of hydrogen bonds at the oil–water interface.

To elucidate the mechanism of composite demulsifiers in W/HO emulsions, the interaction between demulsifiers and IAA molecules including hydrogen bonding was systematically investigated, as shown in Figure 8 and Figure 9. Figure 8 depicts the interaction between demulsifier molecules, water molecules, and IAA molecules. Figure 8a,b show the variation curves of the radial distribution function (RDF) with distance. Notably, the RDF between FAP–water and PFPEA–water molecules exhibited a sharp peak at 6.5 nm, while the RDF between PDC–water molecules exhibited a sharp peak at 5.5 nm. It indicates that there were interactions of demulsifiers–water molecules and demulsifiers–IAA molecules. However, the peak for PDC–water interactions was significantly sharper than those for (1) IAA–water and (2) PDC–IAA interactions. Furthermore, PDC exhibited sharper RDF peaks with both water and IAA molecules compared to FAP and PFPEA, indicating its superior interfacial activity. Moreover, FAP exhibited interaction energies of −6.95 kJ/mol with water molecules and −6.55 kJ/mol with IAA molecules (Figure 8c), while PFPEA showed interaction energies of −7.46 kJ/mol with water molecules and −7.20 kJ/mol with IAA molecules (Figure 8d). Notably, PDC displayed the most robust interactions, with interaction energies reaching −8.59 kJ/mol for water and −7.83 kJ/mol for IAA. These results indicate that the competition between PDC and IAA was the strongest, and the interaction between PDC and water molecules was the strongest. Figure 9a–d show the results of different demulsifiers replacing IAA at the oil–water interface after undergoing all-atom molecular dynamics simulation calculations (simulation time is 500 ps). Although FAP, PFPEA, and PDC all replaced IAA molecules from the oil–water interface, PDC completely transferred displaced IAA molecules into the oil phase, whereas FAP and PFPEA only partially achieved this transition. These results indicate that the synergistic combination of FAP and PFPEA significantly enhanced interfacial film disruption, thus accelerating demulsification of W/HO emulsions. Moreover, Figure 9e shows the variation in the number of hydrogen bonds at the oil–water interface (within a certain statistical range). It can be seen that IAA can form 16 hydrogen bonds at the oil–water interface, mainly composed of N, O, and S atoms in IAA molecules and H atoms in water molecules. When different demulsifiers replace IAA molecules, adding FAP or PFPEA alone can reduce the number of hydrogen bonds between IAA molecules and water molecules at the oil–water interface. FAP formed 28 hydrogen bonds with hydrogen atoms in water molecules through the oxygen atoms in its hydroxyl and ester groups, reducing the number of hydrogen bonds of IAA molecules and water molecules to 7. Meanwhile, PFPEA formed 46 hydrogen bonds with hydrogen atoms in water molecules through its fluorine atoms, lowering the number of hydrogen bonds of IAA molecules and water molecules to 4. However, the combination of FAP and PFPEA completely eliminated hydrogen bonding between IAA and water molecules at the interface. In contrast, PDC maintained more interfacial hydrogen bonds with water molecules than either FAP or PFPEA individually. It was due to the hydrogen bonding formed between the O and F atoms in the complex demulsifier and the H in the water molecule. The results show that the PDA obtained by combining FAP and PFPEA can significantly enhance the hydrogen bond interaction between the demulsifier and water molecules. It is conducive to rapidly break the hydrogen bond and π–π stacking of IAA molecules at the oil–water interface, thus realizing the rapid demulsification of W/HO emulsions [28].

**Figure 10:**
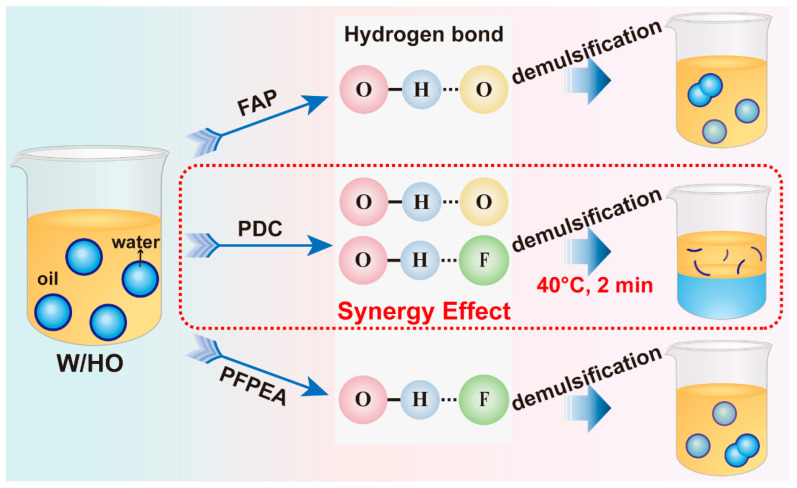
Schematic diagram of the demulsification mechanism.

To sum up, as shown in Figure 10, firstly, the compound demulsifier with higher interfacial activity diffused and rapidly migrated from the oil phase to the oil–water interface. Then, the compound demulsifier rapidly replaced the IAA molecule at the oil–water interface through its stronger interaction with IAA molecules and hydrogen bonding, thus promoting the aggregation and coalescence of water molecules. It is the hydrogen bonding synergistic effect of the compound demulsifier that finally realizes the rapid demulsification of W/HO emulsions.

## 4. Conclusions

The polyether demulsifier complex (PDC) was successfully prepared via physical blending and demonstrated exceptional demulsification performance in IAA-stabilized W/HO emulsions. PDC exhibited a pronounced synergistic effect, outperforming individual demulsifier components. PDC achieved complete demulsification at 400 ppm and 60 °C within 2 min; even at a reduced temperature of 40 °C, complete oil–water separation was attained within 6 min. Research on the demulsification mechanism revealed that PDC with higher interfacial activity enables rapid diffusion and migration from the oil phase to the oil–water interface, displacing IAA molecules through strong interactions with water. This process accelerates water droplet coalescence and phase separation. These findings elucidate the synergistic mechanism of compound demulsifiers and provide critical insights for designing high-efficiency formulations, particularly for low-temperature applications in the petroleum industry. Compared to conventional single-component demulsifiers, PDC offers superior control over interfacial stability and demulsification kinetics under challenging conditions, highlighting its potential for advancing next-generation demulsifier technologies.

## Figures and Tables

**Table 1:** The GPC analysis results of demulsifiers.

Demulsifiers	M_w_ (g/mol)	M_n_ (g/mol)	M_w_/M_n_
FAP	5215	3754	1.39
PFPEA	1264	985	1.28

**Table 2:** Comparison of demulsification performance of demulsifiers.

Demulsifiers	Emulsions	Dosage/ppm	Temperature/°C	Time	Demulsification Efficiency/%	[Ref]
JMNP	water/heavy oil	400	60	30 min	100	[13]
MJTJU-2	water/heavy oil	400	60	15 min	97	[14]
TJU-3	water/crude oil	400	60	25 min	100	[25]
PPBH	water/crude oil	250	40	2 h	96.34	[24]
RBF	water/crude oil	50	60	2 h	95.64	[15]
PGE-TFA	water/crude oil	500	40	90 min	95.53	[26]
GTE-DDA	water/crude oil	400	50	30 min	87.55	[27]
PDC	water/heavy oil	400	40	6 min	100	This work

## Data Availability

The original contributions presented in this study are included in the article/Supplementary Material. Further inquiries can be directed to the corresponding authors.

## Supplementary Materials

The following supporting information can be downloaded at https://www.mdpi.com/article/10.3390/molecules30122550/s1. Figure S1: (a) Oil-water interface model (Blue beads represent water molecules, and red beads represent toluene molecules), (b) mesoscopic molecular model; Table S1: The atomic clusters represented by each bead and repulsive force parameters between the beads (IAA, FAP, toluene and water); Table S2: The atomic clusters represented by each bead and repulsive force parameters between the beads (IAA, PFPEA, toluene and water); Table S3: The atomic clusters represented by each bead and repulsive force parameters between the beads (IAA, PDC, toluene and water); Figure S2: The energy of the molecular structure model varies with the number of simulation steps (a: water, b: toluene, c: IAA, d: FAP, e: PFPEA, f: oil-water interface model); Figure S3: The curve of (a) energy and (b) temperature change with simulation time in the all-atomic molecular simulation system; Figure S4: The change of system energy and temperature with time: energy: (a) FAP, (c) PFPEA, (e) PDC; temperature: (b) FAP, (d) PFPEA, (f) PDC. References [21,29] are cited in the supplementary materials.

## References

[1] Lu T., Li Z., Wang H., Gu Z., Du L. (2023). Reducing CO_2_ emissions and improving oil recovery through silica aerogel for heavy oil thermal production. J. Clean Prod..

[2] Zhao F., Tian Z., Yu Z., Shang H., Wu Y., Zhang Y. (2020). Research status and analysis of stabilization mechanisms and demulsification methods of heavy oil emulsions. Energy Sci. Eng..

[3] Qiao P., Harbottle D., Tchoukov P., Wang X., Xu Z. (2017). Asphaltene subfractions responsible for stabilizing water-in-crude oil emulsions. Part 3. Effect of solvent aromaticity. Energy Fuels.

[4] Fakher S., Ahdaya M., Elturki M., Imqam A. (2020). Critical review of asphaltene properties and factors impacting its stability in crude oil. J. Pet. Explor. Prod. Technol..

[5] Sun N., Hu J., Ma Y., Dong H. (2024). Study on the effect of polymer-modified magnetic nanoparticles on viscosity reduction of heavy oil emulsion. ACS Omega.

[6] Martinez-Palou R., Mosqueira M.d.L., Zapata-Rendon B., Mar-Juarez E., Bernal-Huicochea C., de la Cruz Clavel-Lopez J., Aburto J. (2011). Transportation of heavy and extra-heavy crude oil by pipeline: A review. J. Pet. Sci. Eng..

[7] Kovalenko E.Y., Sagachenko T.A., Cherednichenko K.A., Gerasimova N.N., Cheshkova T.V., Min R.S. (2023). Structural organization of asphaltenes and resins and composition of low polar components of heavy oils. Energy Fuels.

[8] Yang F., Tchoukov P., Pensini E., Dabros T., Czarnecki J., Masliyah J., Xu Z. (2014). Asphaltene subfractions responsible for stabilizing water-in-crude oil emulsions. Part 1: Interfacial behaviors. Energy Fuels.

[9] He C., Zhang X., He L., Sui H., Li X. (2022). Revealing the non-covalent interactions between oxygen-containing demulsifiers and interfacially active asphaltenes: A multi-level computational simulation. Fuel.

[10] Zhang X., Wang J., Zhang X., He L., Sui H., Li X. (2023). Stability of asphaltene-mircoparticles co-stabilized emulsions by oxygen-enriched nonionic demulsifier. J. Mol. Liq..

[11] Ma J., Yao M., Yang Y., Zhang X. (2022). Comprehensive review on stability and demulsification of unconventional heavy oil-water emulsions. J. Mol. Liq..

[12] Hassanshahi N., Hu G., Li J. (2020). Application of ionic liquids for chemical demulsification: A review. Molecules.

[13] Li X., Ma J., Bian R., Cheng J., Sui H., He L. (2020). Novel polyether for efficient demulsification of interfacially active asphaltene-stabilized water-in-oil emulsions. Energy Fuels.

[14] Ma J., Li X., Zhang X., Sui H., He L., Wang S. (2020). A novel oxygen-containing demulsifier for efficient breaking of water-in-oil emulsions. Chem. Eng. J..

[15] Zhang L., Wei L., Shi L., Dai X., Guo S., Jia X., Liu C. (2022). Synthesis and characterization of a novel reticulated multi-branched fluorinated polyether demulsifier for w/o emulsion demulsification. J. Polym. Res..

[16] Ma G., Wang J., He L., Li X., Sui H. (2020). The nature of the Indonesian carbonate asphalt rocks and its insights into the separation processes. J. Pet. Sci. Eng..

[17] Sui H., Ma G., He L., Zhang Z., Li X. (2016). Recovery of heavy hydrocarbons from Indonesian carbonate asphalt rocks. Part 1: Solvent extraction, particle sedimentation, and solvent recycling. Energy Fuels.

[18] Wu J., Xu Y., Dabros T., Hamza H. (2003). Effect of demulsifier properties on destabilization of water-in-oil emulsion. Energy Fuels.

[19] Song X., Shi P., Duan M., Fang S., Ma Y. (2015). Investigation of demulsification efficiency in water-in-crude oil emulsions using dissipative particle dynamics. RSC Adv..

[20] Hu C., Liu S., Fang S., Xiang W., Duan M. (2018). Dissipative particle dynamics investigation of demulsification process and mechanism of comb-like block polyether. Polym. Adv. Technol..

[21] Wang S., Yang S., Wang R., Tian R., Zhang X., Sun Q., Liu L. (2018). Dissipative particle dynamics study on the temperature dependent interfacial tension in surfactant-oil-water mixtures. J. Pet. Sci. Eng..

[22] Ma J., Yang Y., Li X., Sui H., He L. (2021). Mechanisms on the stability and instability of water-in-oil emulsion stabilized by interfacially active asphaltenes: Role of hydrogen bonding reconstructing. Fuel.

[23] Wang D., Lin M., Dong Z., Li L., Jin S., Pan D., Yang Z. (2016). Mechanism of high stability of water-in-oil emulsions at high temperature. Energy Fuels.

[24] Qu Q., Li H., Li S., Hu Z., Zhu M., Chen J., Sun X., Tang Y., Zhang Z., Mi Y. (2023). Synthesis and demulsification mechanism of an ionic liquid with four hydrophobic branches and four ionic centers. Chemosphere.

[25] Zhang X., He C., Zhou J., Tian Y., He L., Sui H., Li X. (2023). Demulsification of water-in-heavy oil emulsions by oxygen-enriched non-ionic demulsifier: Synthesis, characterization and mechanisms. Fuel.

[26] Lei M., Huang H., Liu J., Peng F. (2023). A gemini ionic liquid and its low-temperature demulsification performance in water-in-crude oil emulsions. Colloid Surf. A-Physicochem. Eng. Asp..

[27] Zhang Z., Ai G., Zeng G., Yuan H., Yang Y., Shen L., Feng X., Ye F., Mi Y. (2022). Demulsification of water-in-crude oil emulsion driven by a three-branch structure demulsifier. J. Mol. Liq..

[28] Tian Y., He C., He L., Xu Z., Sui H., Li X. (2024). Doping heteroatoms to form multiple hydrogen bond sites for enhanced interfacial reconstruction and separations. J. Hazard. Mater..

[29] Geng X., Li C., Zhang L., Guo H., Shan C., Jia X., Wei L., Cai Y., Han L. (2022). Screening and demulsification mechanism of fluorinated demulsifier based on molecular dynamics simulation. Molecules.

