# Supplementary material for: Polyether Demulsifier Complexes for Efficient Demulsification of Water-in-Heavy Oil Emulsions
Source: Molecules. 2025 Jun 11;30(12):2550. doi: 10.3390/molecules30122550 (PMC12195951; doi:10.3390/molecules30122550)
Supplement: Supplementary file 1 [file molecules-30-02550-s001.zip › molecules-3638008-supplementary.pdf]

Polyether demulsifier complexes for efficient demulsification of water-in-heavy oil emulsions

Jing Li [1,†], Xiao Xia [2,†], Jinlong Gao [1], Hao Chen [3] and Jun Ma [2,*]

[1]*Department of Chemical Engineering, Textile and Clothing, Shaanxi Polytechnic Institute, Xianyang, Shanxi 712000, China,*

[2]*Department of Chemical Engineering, School of Chemistry and Chemical Engineering, Guizhou University, Guiyang, Guizhou 550025, China*

[3]*China Tianchen Engineering Corporation, Tianjin 300400, China*

[*]Corresponding author: Jun Ma

E-mail: Junma_@tju.edu.cn

[†]These authors contributed equally to this work.

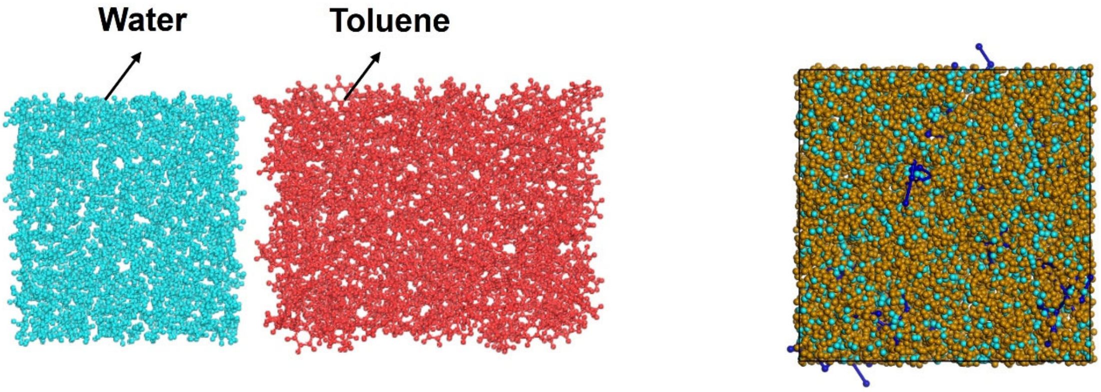

(a) **(b)**

Figure S1. (a) Oil-water interface model (Blue beads represent water molecules, and red beads represent toluene molecules), (b) mesoscopic molecular model

Table S1. The atomic clusters represented by each bead and repulsive force parameters between the beads (IAA, FAP, toluene and water).

Automic clusters	Beads	a1	b1	c1	d1	e1	f1	g1	h1	A	B	C	D	E	T1	T2	W
$C_{14}H_1$	a1	25	108	101	70	116	116	30	89	37	92	92	89	42	133	36	197
C_6H_{13}	b1	108	25	28	148	25	25	154	25	36	31	31	30	45	27	25	73
C_6H_{12}	c1	101	28	25	105	28	28	128	28	35	31	31	30	41	30	25	73
$C_{15}H_1$	d1	70	148	105	25	140	140	28	105	41	89	89	88	48	140	35	180
$C_{15}H_{11}$	e1	116	25	28	140	25	25	102	25	25	31	31	30	27	27	25	73
C_5H_{10}	f1	116	25	28	140	25	25	102	25	35	31	31	30	45	27	25	73
$C_{14}H_2$	g1	30	154	128	28	102	102	25	91	38	118	118	116	44	134	67	185
C_7H_{15}	h1	89	25	28	105	25	25	91	25	35	32	32	31	45	28	25	73
C_3H_7O	A	33	35	35	41	25	35	37	36	25	30	30	29	26	26	36	72
C_2H_2O	B	92	31	31	89	31	31	118	32	30	25	25	25	29	27	32	45
C_2H_2O	C	92	31	31	89	31	31	118	32	30	25	25	25	29	27	32	45
C_3H_4O	D	89	30	30	88	30	30	116	31	29	25	25	25	29	26	31	45
C_3H_5O	E	42	45	41	48	27	45	44	45	26	29	29	29	25	31	45	25
C_6H_5	T1	133	27	30	140	27	27	134	28	26	27	27	26	31	25	34	151
CH_3	T2	36	25	25	35	25	25	67	25	36	32	32	31	45	34	25	151
H_6O_3	W	197	73	73	180	73	73	185	73	72	45	45	25	25	151	151	25

Table S2. The atomic clusters represented by each bead and repulsive force parameters between the beads (IAA, PFPEA, toluene and water)

Automic clusters	Beads	a1	b1	c1	d1	e1	f1	g1	h1	a2	b2	c2	d2	e2	f2	T1	T2	W
$C_{14}H_1$	a1	25	108	101	70	116	116	30	89	40	37	40	38	42	43	133	36	197
C_6H_{13}	b1	108	25	28	148	25	25	154	25	26	29	28	27	27	36	27	25	73
C_6H_{12}	c1	101	28	25	105	28	28	128	28	26	29	28	27	27	35	30	25	73
$C_{15}H_1$	d1	70	148	105	25	140	140	28	105	37	35	36	36	39	45	140	35	180
$C_{15}H_{11}$	e1	116	25	28	140	25	25	102	25	27	28	27	27	28	30	27	25	73
C_5H_{10}	f1	116	25	28	140	25	25	102	25	26	29	28	27	27	36	27	25	73
$C_{14}H_2$	g1	30	154	128	28	102	102	25	91	43	43	44	42	45	46	134	67	185
C_7H_{15}	h1	89	25	28	105	25	25	91	25	27	29	28	27	28	36	28	25	73
C_2F_5	a2	40	26	26	37	27	26	43	27	25	25	25	25	25	29	29	26	36
C_2OF_4	b2	37	29	29	35	28	29	43	29	25	25	25	25	25	30	25	29	39
C_2OF_2	c2	40	28	28	36	27	28	44	28	25	25	25	25	25	29	28	28	37
C_3OF_4	d2	38	27	27	36	27	27	42	27	25	25	25	25	25	28	28	27	35
C_2F_4	e2	42	27	27	39	28	27	45	28	25	25	25	25	25	30	27	27	38
CH_3O	f2	43	36	35	45	30	36	46	36	29	30	29	28	30	25	26	35	31
C_6H_5	T1	133	27	30	140	27	27	134	28	29	25	28	28	27	26	25	34	151
CH_3	T2	36	25	25	35	25	25	67	25	26	29	28	27	27	35	34	25	151
H_6O_3	W	197	73	73	180	73	73	185	73	36	39	37	35	38	31	151	151	25

Table S3. The atomic clusters represented by each bead and repulsive force parameters between the beads (IAA, PDC, toluene and water)

Automic clusters	Beads	a1	b1	c1	d1	e1	f1	g1	h1	A	B	C	D	E	a2	b2	c2	d2	e2	f2	T1	T2	W
$C_{14}H_1$	a1	25	108	101	70	116	116	30	89	37	92	92	89	42	40	37	40	38	42	43	133	36	197
C_6H_{13}	b1	108	25	28	148	25	25	154	25	36	31	31	30	45	26	29	28	27	27	36	27	25	73
C_6H_{12}	c1	101	28	25	105	28	28	128	28	35	31	31	30	41	26	29	28	27	27	35	30	25	73
$C_{15}H_1$	d1	70	148	105	25	140	140	28	105	41	89	89	88	48	37	35	36	36	39	45	140	35	180
$C_{15}H_{11}$	e1	116	25	28	140	25	25	102	25	25	31	31	30	27	27	28	27	27	28	30	27	25	73
C_5H_{10}	f1	116	25	28	140	25	25	102	25	35	31	31	30	45	26	29	28	27	27	36	27	25	73
$C_{14}H_2$	g1	30	154	128	28	102	102	25	91	38	118	118	116	44	43	43	44	42	45	46	134	67	185
C_7H_{15}	h1	89	25	28	105	25	25	91	25	35	32	32	31	45	27	29	28	27	28	36	28	25	73
C_3H_7O	A	33	35	35	41	25	35	37	36	25	30	30	29	26	29	28	28	28	29	26	26	36	72
C_2H_2O	B	92	31	31	89	31	31	118	32	30	25	25	25	29	28	26	27	29	27	26	27	32	45
C_2H_2O	C	92	31	31	89	31	31	118	32	30	25	25	25	29	28	26	27	29	27	26	27	32	45
C_3H_4O	D	89	30	30	88	30	30	116	31	29	25	25	25	29	28	25	27	29	27	26	26	31	45
C_3H_5O	E	42	45	41	48	27	45	44	45	26	29	29	29	25	32	34	33	30	33	27	31	45	25
C_2F_5	a2	40	26	26	37	27	26	43	27	29	28	28	28	32	25	25	25	25	25	29	29	26	36
C_2OF_4	b2	37	29	29	35	28	29	43	29	28	26	26	25	34	25	25	25	25	25	30	25	29	39
C_2OF_2	c2	40	28	28	36	27	28	44	28	28	27	27	27	33	25	25	25	25	25	29	28	28	37
C_3OF_4	d2	38	27	27	36	27	27	42	27	28	29	29	29	30	25	25	25	25	25	28	28	27	35
C_2F_4	e2	42	27	27	39	28	27	45	28	29	27	27	27	33	25	25	25	25	25	30	27	27	38
CH_3O	f2	43	36	35	45	30	36	46	36	26	26	26	26	27	29	30	29	28	30	25	26	35	31
C_6H_5	T1	133	27	30	140	27	27	134	28	26	27	27	26	31	29	25	28	28	27	26	25	34	151
CH_3	T2	36	25	25	35	25	25	67	25	36	32	32	31	45	26	29	28	27	27	35	34	25	151
H_6O_3	W	197	73	73	180	73	73	185	73	72	45	45	45	25	36	39	37	35	38	31	151	151	25

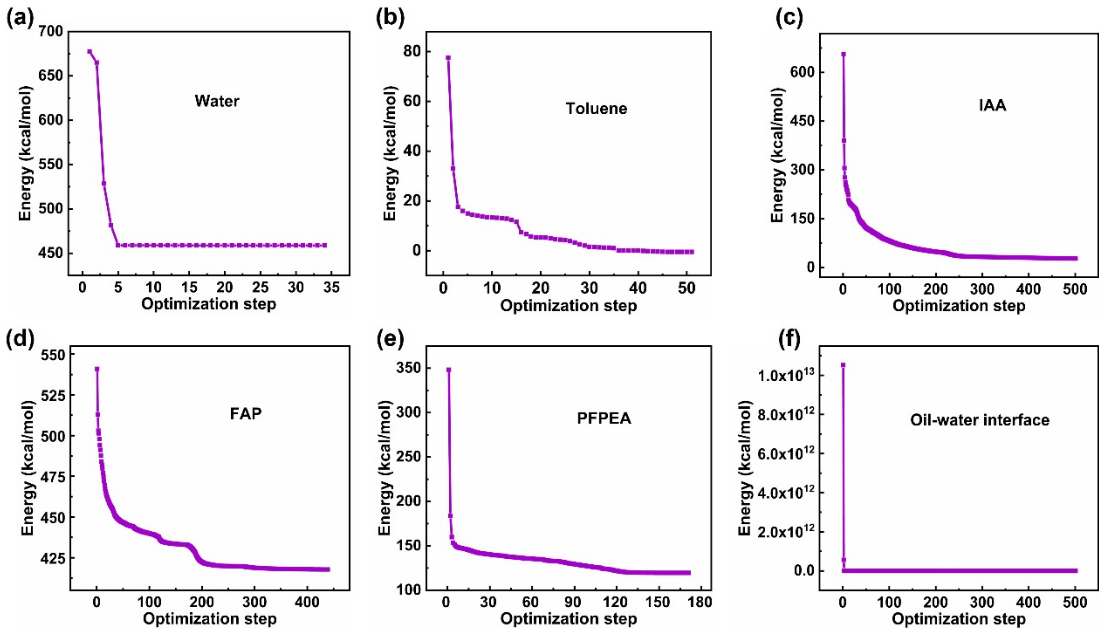

Figure S2. The energy of the molecular structure model varies with the number of simulation steps (a: water, b: toluene, c: IAA, d: FAP, e: PFPEA, f: oil-water interface model).

To explore the action mechanism of IAA at the oil-water interface, an all-atom molecular dynamics simulation was carried out. **Figure S3** shows the change curves of energy and temperature in the simulation system with the simulation time. As can be seen from the figure, the curves of energy and temperature change frequently with the simulation time. However, with the extension of the simulation time, the fluctuation range error of energy and temperature is within the normal error range, indicating that the system reaches the equilibrium state [1].

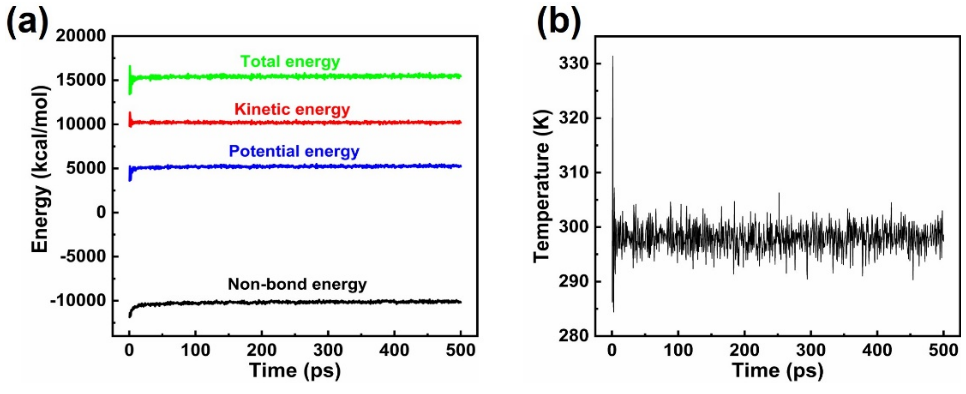

Figure S3. The curve of (a) energy and (b) temperature change with simulation time in

the all-atomic molecular simulation system.

Figure S4 shows the change of energy and temperature of the simulation system with the simulation time during the DPD simulation calculation process. It can be seen from **Figures S4a** and **S4b** that with the extension of simulation time, the simulated change curves of energy and temperature of FAP fluctuate frequently, but the fluctuation error is within the normal range, indicating that the simulation time of 9000 ps meets the requirements of the dynamic calculation simulation process, and the dynamic system of FAP reaches an equilibrium state. Similarly, **Figures S4c** and **S4d** show that the dynamic system of PFPEA reaches an equilibrium state at 7500 ps simulation time, and the dynamic system of PDC at 4000 ps simulation time at **Figures S4e** and **S4f** surface reaches an equilibrium state [2].

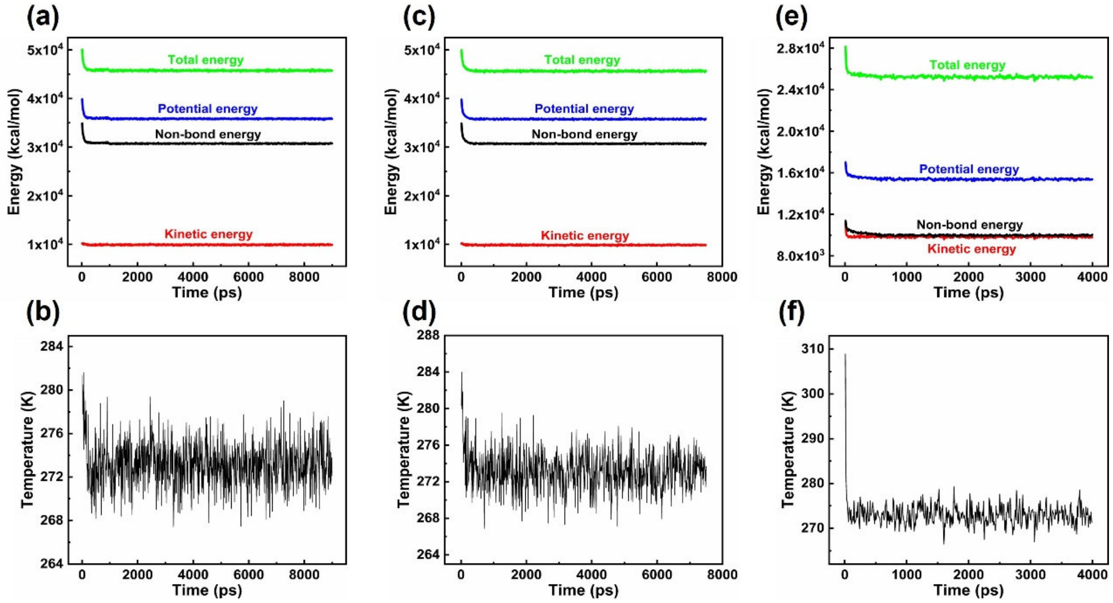

Figure S4. The change of system energy and temperature with time: energy: (a) FAP, (c) PFPEA, (e) PDC; temperature: (b) FAP, (d) PFPEA, (f) PDC.

References

[1] X. Geng, C. Li, L. Zhang, H. Guo, C. Shan, X. Jia, L. Wei, Y. Cai, L. Han, Screening and demulsification mechanism of fluorinated demulsifier based on molecular dynamics simulation, Molecules, 27 (2022) 1799.

[2] S. Wang, S. Yang, R. Wang, R. Tian, X. Zhang, Q. Sun, L. Liu, Dissipative particle dynamics study on the temperature dependent interfacial tension in surfactant-oil-water mixtures, J. Pet. Sci. Eng. 169 (2018) 81-95.
